# DjinniChip: evaluation of a novel molecular rapid diagnostic device for the detection of *Chlamydia trachomatis* in trachoma-endemic areas

**DOI:** 10.1186/s13071-020-04414-6

**Published:** 2020-10-27

**Authors:** Tamsyn R. Derrick, Natalia Sandetskaya, Harry Pickering, Andreas Kölsch, Athumani Ramadhani, Elias Mafuru, Patrick Massae, Aiweda Malisa, Tara Mtuy, Matthew J. Burton, Martin J. Holland, Dirk Kuhlmeier

**Affiliations:** 1 Clinical Research Department, London School of Hygiene and Tropical Medicine, London, UK; 2 Eye Health Project, Kilimanjaro Christian Medical Centre, Moshi, Tanzania; 3 Department of Diagnostics, Fraunhofer Institute Cell Therapy and Immunology IZI, Leipzig, Germany

**Keywords:** *Chlamydia trachomatis*, Trachoma, LAMP, Rapid test, Lateral flow assay

## Abstract

**Background:**

The clinical signs of active trachoma are often present in the absence of ocular *Chlamydia trachomatis* infection, particularly following mass drug administration. Treatment decisions following impact surveys and in post-control surveillance for communities are currently based on the prevalence of clinical signs, which may result in further unnecessary distribution of mass antibiotic treatment and the increased spread of macrolide resistance alleles in ‘off-target’ bacterial species. We therefore developed a simple, fast, low cost diagnostic assay (DjinniChip) for diagnosis of ocular *C. trachomatis* for use by trachoma control programmes.

**Methods:**

The study was conducted in the UK, Germany and Tanzania. For clinical testing in Tanzania, specimens from a sample of 350 children between the ages of 7 to 15 years, which were part of a longitudinal cohort that began in February 2012 were selected. Two ocular swabs were taken from the right eye. The second swab was collected dry, kept cool in the field and archived at – 80 °C before sample lysis for DjinniChip detection and parallel nucleic acid purification and detection/quantification by qPCR assay.

**Results:**

DjinniChip was able to reliably detect > 10 copies of *C. trachomatis* per test and correctly identified 7/10 Quality Control for Molecular Diagnostics *C. trachomatis* panel samples, failing to detect 3 positive samples with genome equivalent amounts ≤ 10 copies. DjinniChip performed well across a range of typical trachoma field conditions and when used by lay personnel using a series of mock samples. In the laboratory in Tanzania, using clinical samples the sensitivity and specificity of DjinniChip for *C. trachomatis* was 66% (95% CI 51–78) and 94.8 (95% CI 91–97%) with an overall accuracy of 90.1 (95% CI 86.4–93).

**Conclusions:**

DjinniChip performance is extremely promising, particularly its ability to detect low concentrations of *C. trachomatis* and its usability in field conditions. The DjinniChip requires further development to reduce inhibition and advance toward a closed system. DjinniChip results did not vary between local laboratory results and typical trachoma field settings, illustrating its potential for use in low-resource areas to prevent unnecessary rounds of MDA and to monitor for *C. trachomatis* recrudescence. 
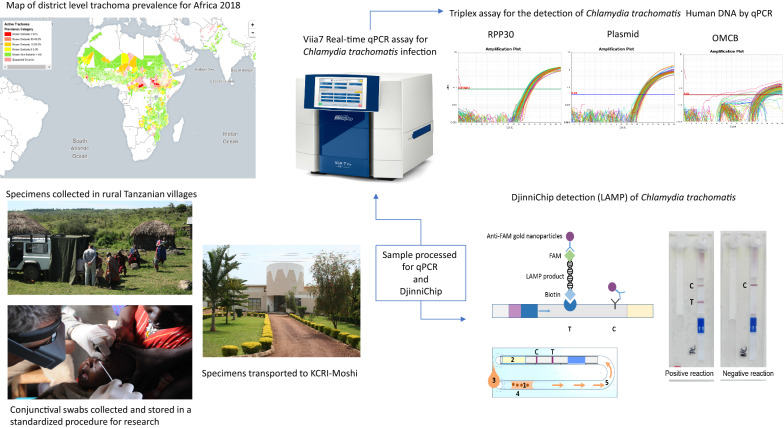

## Background

Globally, approximately 40 million people, mostly children, are thought to have active trachoma at any given time [1]. The World Health Organization (WHO) recommends community-based mass drug administration (MDA) coupled with facial hygiene and environmental improvements (key components of the SAFE strategy (Surgery for trichiasis, Antibiotics, Facial cleanliness, Environmental improvements)) in districts where the prevalence of the clinical sign of trachomatous inflammation-follicular (TF) is ≥ 10% among children aged 1–9 years. However, a critical issue for control programmes is the poor correlation between disease signs and infection particularly after MDA; the long-term persistence of clinical signs leads to uncertainty about the impact of programmes [2].

Currently, ocular infection testing is not recommended by the WHO as an indicator for national trachoma programmes. There is continued need for low-cost, simple to use tests for *Chlamydia trachomatis* to guide district-level treatment decisions, particularly around whether MDA can be discontinued, and to support the WHO-led process to validate elimination as a public health problem in formerly endemic regions. In order to be suitably sensitive and specific, the diagnostic test should detec*t C. trachomatis* nucleic acid, require minimal sample preparation and testing should be performed at a district-level laboratory [3, 4]. Several nucleic acid amplification (NAAT) tests are available which have excellent sensitivity and specificity (> 95%) for chlamydial infections, including the Cepheid Xpert CT/NG test (result in 90 min) and the Atlas Genetics io^®^ CT/NG test (result in 30 min) which require minimal sample preparation [5, 6]. Both however require specialised proprietary instruments and a robust alternating-current electric power supply form a national utility grid or local generator. Each are cost-prohibitive (> US$10/test) and the throughput for programmatic use may be insufficient.

A novel isothermal NAAT diagnostic device for *C. trachomatis* detection was developed; “DjinniChip”, intended to be utilised by trachoma surveillance and control programmes. The test was designed to be performed primarily at the district level laboratory by lay personnel and requires only a portable heat block, able to be powered by 12V car battery, with a total test time of approximately 50 min. The approximate cost per test after scale-up is < US$2; however, the test is not yet available commercially and its performance has not been formally evaluated. The aim of this study was to perform a preliminary evaluation of the performance of the DjinniChip from archived ocular clinical samples in a trachoma-endemic region in-country, initially in the controlled environment of a research laboratory. The secondary aim was to determine the DjinniChip’s resilience and usability when used in typical conditions and facilities where trachoma is endemic and by lay personnel.

## Methods

### Study design, participants and sample size

A retrospective study was conducted using archived conjunctival swab samples collected from children in trachoma-endemic communities in northern Tanzania to assess the performance of the DjinniChip. In February 2012, we recruited a cohort of children from three adjacent trachoma endemic villages in Kilimanjaro and Arusha regions, northern Tanzania. All children, between the ages 3 and 11 years, who were normally resident in one of the three villages, were eligible for inclusion (*n* = 666) [7]. A total of 616 children were enrolled and the cohort was initially followed-up every 3 months for 4 years, for a total of 17 time-points. Two conjunctival swab samples were collected consecutively from the right eye at each time-point. Time-point 14 (August 2015) was selected for this evaluation and 428 children were seen, 9 months after the last mass community treatment with antibiotic as detailed by the SAFE strategy. Conjunctival swabs were collected as previously described using sterile polyester-tipped swabs [7, 8]. The first swab was collected into 250 µl RNA later and was used elsewhere [7–9], whilst the second swab was collected dry and stored at − 80 °C until used in this study. 0.5% of swabs collected were ‘air’ control swabs in order to test for field- and laboratory contamination. These samples were collected by passing a swab 10 cm from a participantʼs everted eye, these were labelled and processed identically to participant samples.

The study sample size was determined by the number of DjinniChips manufactured and shipped to KCRI-BL (Tanzania) for the study (*n* = 600) and the number of participants seen at time-point 14 (*n* = 428). Based on the observed infection prevalence 9 months after MDA treatment round 1 (time-point 7) and 2 (time-point 11) the expected prevalence of ocular infection was estimated at 8%. The precision of the prevalence estimate based on this sample size at the 95% confidence level is 5%. Alternatively, assuming the index test (DjinniChip) achieves sensitivity and specificity of 95%, a sample size of 428 would achieve an odds of diagnosing a true (qPCR) positive of 62% (95% CI 51–72%) and an odds of 1% (95% CI 0–2%) of a false negative.

The WHO endorsed SAFE strategy (surgery for trichiasis (in-turned eyelashes), antibiotics, facial cleanliness, and environmental improvement) was implemented in the study villages as approved by the Tanzanian Ministry of Health (MoH). Education about environmental improvements and facial hygiene was provided by the field team, free trichiasis surgery was offered, and all members of the three villages (including study participants) were offered azithromycin for trachoma control immediately following time-points 3, 7 and 11 specimen collection in August of the years 2012, 2013, and 2014.

### DNA extraction

Dry clinical samples were thawed and 400 µl of lysis-amplification buffer (20 mM Tris-HCl, 10 mM (NH_4_)_2_SO_4_, 150 mM KCl, 2 mM MgSO_4_, 0.1% Tween^®^ 20, 1% Triton X, pH 8.8) was added to the tube. Samples were vortexed for 30 s, pulse-centrifuged, and incubated for 10 min at 95 °C for sample lysis. Lysates were then pulse-centrifuged and incubated at room temperature for 10 min. For the positive control (PC), 46 µl of lysis-amplification buffer was spiked with 4 µl *C. trachomatis* DNA (10,000 omcB copies/µl) derived from *C. trachomatis* L2/434 which was originally cultured and prepared at the London School of Hygiene & Tropical Medicine (LSHTM). For the negative control (NC), 50 µl of lysis-amplification buffer was used. One hundred microliters of sample lysate was used for DNA extraction and *C. trachomatis* detection by quantitative PCR (qPCR) and 25 µl was used for *C. trachomatis* detection by DjinniChip (see below). Remaining sample lysate was stored at − 20 °C.

### qPCR detection of *C. trachomatis*

One hundred microliters of sample lysate was mixed 1:1 with 100 µl of sterile PBS and DNA was extracted using the QIAamp DNA mini kit (Qiagen Ltd, Manchester, UK) following the manufacturer’s instructions. DNA was eluted into 60 µl of buffer AE and stored at 4 °C prior to qPCR testing. *Chlamydia trachomatis* was detected using a reference qPCR assay, which has previously been evaluated against droplet digital PCR and Artus (Qiagen Ltd, Manchester, UK) *C. trachomatis* diagnostic assays for ocular samples [10]. The triplex qPCR assay detects chlamydial chromosomal (omcB) and plasmid (pORF2) targets and a human endogenous control gene (RPP30). Samples were tested in duplicate, with each 20 µl reaction containing 1× TaqMan Multiplex Master Mix (Thermo Fisher Scientific, Inchinnan, UK), 300 nM of all primers and probes and 4 µl DNA. Samples were considered *C. trachomatis* positive if RPP30 in combination with pORF2 and/or omcB targets amplified in < 40 cycles in either or both replicates. qPCR data were processed in STATA v15 and target concentrations were calculated by extrapolating from a standard curve. Clinical information from participants at time-point 14 was available to the assessors of the reference qPCR test but the result of the index test was masked until the laboratory study was completed.

### Droplet digital PCR (ddPCR) detection of *C. trachomatis*

ddPCR was used to detect and quantify *C. trachomatis* infectious load in 12 archived conjunctival clinical samples and in Quality Control for Molecular Diagnostics (QCMD, Glasgow, UK) panel samples for the preliminary evaluation of DjinniChips. Dry, frozen conjunctival swabs from 4 *C. trachomatis*-negative and 8 *C. trachomatis*-positive individuals, previously determined by an in-house *16S* rRNA qPCR [11], were incubated for 10 min at room temperature in 400 µl elution buffer (1× PBS or 1% Triton X-100 + 10 mM Tris-HCl (pH7)). 200 µl of eluate was frozen for DjinniChip evaluation. DNA was extracted from the remaining 200 µl using the Blood and Serum DNA Isolation Kit (BioChain Institute Inc, Newark, CA, USA). For QCMD samples, DNA was extracted using the QIAamp Mini DNA Extraction (Qiagen Ltd, Manchester, UK). *Chlamydia trachomatis* plasmid and genome were quantified as previously described [10].

### Preparation of DjinniChips

DjinniChip is an integrated diagnostic platform which amplifies DNA from the chromosomal porB gene of *C. trachomatis* using loop-mediated isothermal amplification (LAMP). The chips were produced by injection-moulding of cyclic olefin copolymer in Kunststoff-Zentrum in Leipzig (Germany). The reagents for LAMP were freeze-dried in the channel of the chip. Each portion of reaction mix contained 300 nmol MgSO_4 ,_ 16 U glycerol-free Bst 3.0 polymerase (New England Biolabs (Frankfurt am Main, Germany), 25 mmol of dA, dG, dC, 22.5 mmol of dU, 0.5 mmol dT (Jena Bioscience, Jena, Germany), 10 pmol of primers B3 and F3, 80 pmol of primer BIP, 60 pmol of unlabelled primer FIP, 20 pmol of 5'-biotin-labelled FIP, 40 pmol of 5'-FAM-labelled primer LB and 40 pmol of 5'-biotin-labelled primer LF (Eurofins Genomics, Ebersberg, Germany), 0.25 mg/ml (w/v) BSA and 6% trehalose (Sigma Aldrich, Taufkirchen, Germany). The primer sequences are provided in Table 1. The freeze-dried reagents were overlaid with a 4.3 × 35 mm PES filter membrane (Merck Millipore, Darmstadt, Germany), 0.22 µm pore size. A lateral flow test strip DetectLine Basic (Amodia, Braunschweig, Germany) was placed in the chip, and the bottom surface of the chip was sealed with Masterclear^®^ real-time PCR Film (Eppendorf, Hamburg, Germany). DjinniChips were manufactured < 6 weeks prior to testing and were stored in vacuum-sealed sachets at 4 °C until use. The quality of each batch of the chips was verified by testing a positive (0.001 ng *C. trachomatis* DNA) and a negative control on random chips from the batch.

**Table 1:** LAMP primers for the detection of *Chlamydia trachomatis* by DjinniChip

Primer	Sequence (5'–3')
F3	CTCTGCTTCTGCGGAGAT
B3	CAGCGAACGATCAGGAAG
FIP	AGGAGAAGGACCAACCCCAGCTCAGGTAAAAGATGTCCCT
BIP	TTCGACAACCAAAACGCGAAGGAAAAAGCTACAGCTACAC
LF	CTGTTGTCACAGAGGTAACGAC
LB	CGATCTCGTGAACTGTAATCTCAAT

### DjinniChip detection of *C. trachomatis*

**Fig. 1:**
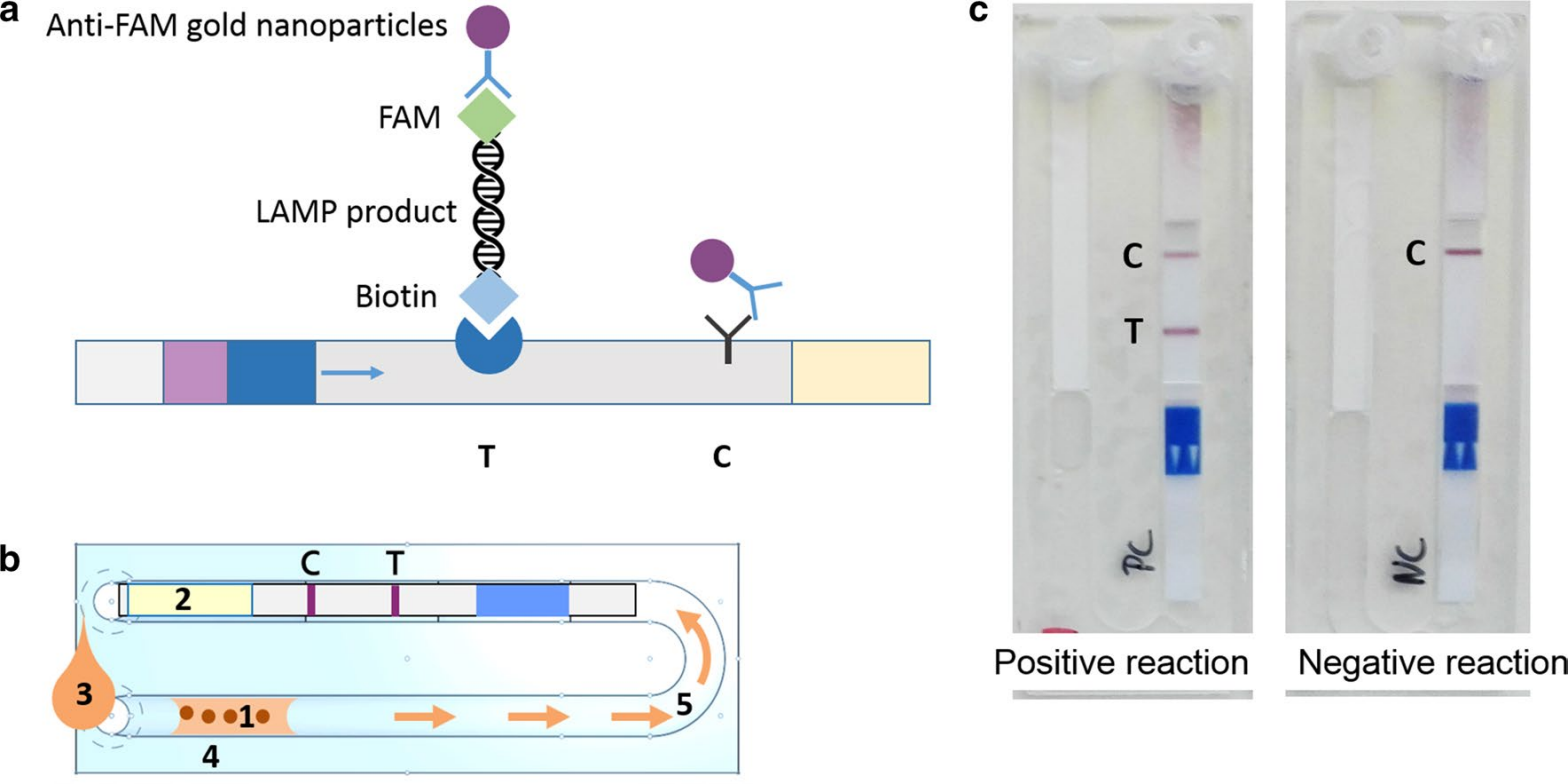
Schematic principle of the DjinniChip. **a** Principle of the detection of LAMP product on LFS. **b** Components and processes. The DjinniChip contains lyophilized reagents (1) and a lateral flow test strip (2). A liquid sample (3) is loaded onto the chip through its inlet. The liquid sample reconstitutes the reagents (4). *Chlamydia trachomatis* DNA is amplified at 65 °C. The reaction mix is pumped gently with a syringe to the lateral flow test (5). The appearance of the Control band (C) indicates the correct function of the lateral flow test. The appearance of the Test band (T) indicates the presence of *C. trachomatis* in the sample**. c** Detection on DjinniChips

Twenty-five microliters of sample lysate (see “DNA extraction” section above) was mixed with 25 µl lysis-amplification buffer and the entire 50 µl was added to the DjinniChip. Sample extract was driven by capillary effect into the reaction channel, dissolving the lyophilized reagents. The inlets of the chip were sealed with Air-O-Seal membrane (4titude, Berlin, Germany). Following incubation on the flat bed of a heat block for 35 min at 65 °C for the LAMP reaction, the reaction was stopped by placing the DjinniChip on a flat ice block (cooling battery) for 2–5 min. The amplified product was then manually pumped towards the integrated lateral flow strip (LFS) by injecting 180 µl chromatographic buffer (Amodia, Braunschweig, Germany) into the sample inlet. Biotin- and fluorescein (FAM)-labelled amplicons allowed LFS detection after 10 min incubation at room temperature, by use of gold nanoparticles attached to anti-fluorescein-antibodies (Fig. 1). A positive control signal (C) and signal at the test zone (T) corresponded to *C. trachomatis* in the patient sample. Results were recorded electronically, and photographic records were taken of each test result.

Samples were analysed using a second DjinniChip if (i) the negative no-template control (NC) was weakly or strongly positive for the analysed batch of samples, or (ii) if the test band was only weakly positive (Additional file 1: Figure S1). In either of these cases positive samples were re-tested, with additional positive and negative controls. The outcome of the second test was used as the final result: if the NC was negative but the sample was weakly positive again, the sample was determined positive. Clinical information and the reference test results were masked to the assessors of the Djinnichip results. The study was conducted and reported according to the STARD guidelines for diagnostic studies (Additional file 1: Table S1) [12].

### Mock samples

In order to determine the resilience and usability of the DjinniChip, 100 mock samples were tested in field conditions. Mock samples consisted of a 50 µl suspension of *C. trachomatis* A2497 elementary bodies at four different concentrations (below) in PBS which were added to sterile polyester-tipped swabs and stored dry in 1.5 ml tubes at − 80 °C for a minimum of 1 week. *Chlamydia trachomatis* A2497 was grown in HEp-2 cells and purified as previously described and quantified by ddPCR [13]. Four field sites were selected: two humid and warm and two hot, dry and dusty. Results were compared to replicate mock samples tested on DjinniChips at Kilimanjaro Clinical Research Institute-Biotechnology Laboratory (KCRI-BL). At each of the five sites, 20 mock samples ranging in *C. trachomatis* concentration were tested (quadruplicates of negative control, 160 copies/swab, 800 copies/swab, 3200 copies/swab, 16,000 copies/swab); 10 duplicates by an experienced laboratory technician and 10 duplicates by a field nurse who received basic training. A heat block (Thermo Fisher Scientific, Inchinnan, UK) powered from a 12V direct-current car battery was used to heat samples at mobile field sites. DjinniChip testing was performed in the open boot of a stationary Land Rover. The field team drove to each field site (selected to provide a range of typical trachoma environmental conditions), where testing was performed in the open boot of the stationary vehicle. Samples were transported on ice in a cool box and reactions were stopped on the surface of flat ice blocks. Results (including photograph), temperature, humidity and time to process 10 tests was recorded. Remaining sample extract was frozen at − 20 °C prior to DNA extraction (Qiagen Ltd, Manchester, UK) and qPCR testing for *C. trachomatis.*

## Results

### Preliminary evaluation

DjinniChip performance was initially tested in a European research and development laboratory (Fraunhofer IZI, Leipzig) using anonymised conjunctival dry swab samples that had been collected and stored in an identical manner from a previous study [14] to that described for time-point 14 in parallel with a QCMD panel. Of the 12 clinical swabs tested, the DjinniChip correctly diagnosed all samples as *C. trachomatis* positive or negative; 8/8 positives and 4/4 negatives (Table 2, Additional file 1: Figure S2). The concentration of the sample with the lowest chlamydial load, correctly identified as positive by the DjinniChip, was 36 genome copies per reaction (corresponding to 573 copies per swab; quantified by ddPCR). The QCMD panel consisted of the extracted DNA of urogenital *C. trachomatis* serovars from swabs and urine. Of the 10 QCMD samples tested, 2/2 negatives and 5/8 positives were correctly diagnosed by the DjinniChip (Table 3, Additional file 1: Figure S3). The three false negatives were the three lowest concentration samples, each at 10 target copies per reaction.

**Table 2:** Evaluation of DjinniChip in eluted ocular swabs

*C. trachomatis* genome copies per swab	*C. trachomatis* genome copies per reaction on chip (sample of 25 µl)	DjinniChip test result
0	0	−
0	0	−
0	0	−
0	0	−
573	36	+
1579	99	+
1794	113	+
6739	421	+
13270	830	+
14989	936	+
185611	11,601	+
627486	39,218	+

DjinniChip samples were tested at the Fraunhofer Institute, Germany. ddPCR was performed at LSHTM, UK

*Key*: +, positive; –, negative

**Table 3:** Evaluation of DjinniChip in QCMD (Quality Control for Molecular Diagnostics) DNA sample panel

Sample code	Sample content	Copies/µl of extracted DNA	Results of DjinniChip test (sample of 10 µl)	Copies/reaction on chip
CTDNA17S-01	*C. trachomatis* (LGV)	3	+	30
CTDNA17S-02	*C. trachomatis* (LGV)	1	– ^a^	10
CTDNA17S-03	*C. trachomatis* (LGV)	11	+	110
CTDNA17S-04	*C. trachomatis* (LGV)	6	+	60
CTDNA17S-05	*C. trachomatis* (Genovar F)	5	+	50
CTDNA17S-06	*C. trachomatis* (LGV)	1	– ^a^	10
CTDNA17S-07	*C. trachomatis* (LGV) + *N. gonorrhoeae* (ST49226)	1	–^a^	10
CTDNA17S-08	Negative	0	–	0
CTDNA17S-09	*C. trachomatis* (Swedish strain)	433	+	4330
CTDNA17S-10	Negative	0	–	0

DjinniChip samples were tested at the Fraunhofer Institute, Germany

LGV, lymphogranuloma venereum

*Key*: +, positive; –, negative

^a^Samples with low copy number identified on DjinniChip as negative

### Evaluation with clinical samples

**Fig. 2:**
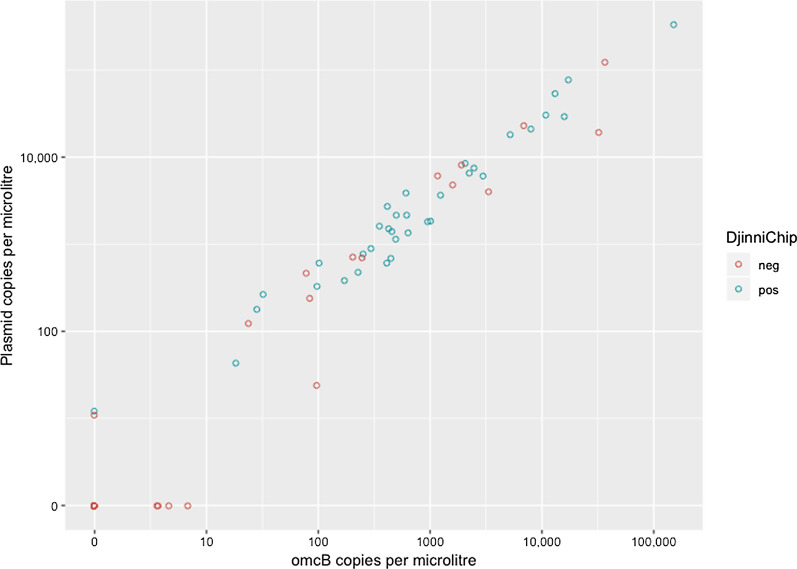
Concentration of *C. trachomatis* positive clinical samples by reference qPCR in target copies per microlitre of extracted DNA, showing those diagnosed as positive (blue circles, pos) and negative (red circles, neg) by the DjinniChip

The demographic and clinical characteristics of the 350 participants retained in the Djinnichip analysis are shown in Table 4. In the field, 430 dry swab specimens were collected (428 clinical samples and 2 ‘air controls’ as detailed in Methods). Of these, 352 dry swab samples were eluted and tested in parallel using the DjinniChip and the reference test (qPCR) at the KCRI-BL research laboratory, Moshi, Tanzania (Table 5, Additional file 1: Table S2). Seventy-eight consecutive dry swab samples (CD101-178) were excluded due to a laboratory error in specimen processing (unrelated to DjinniChip testing). There were 18 DjinniChip false negatives relative to qPCR diagnosis; of these, 9 had high chlamydial load by qPCR, with over 100 copies per µl of extracted DNA (Fig. 2). The performance characteristics of sensitivity, specificity, PPV, NPV and accuracy for all 352 specimens (350 clinical samples and 2 air controls) are shown in Table 6. The Djinnichip identified fewer specimens from those with the clinical signs of trachoma (21 *vs* 30) and tended to identify more specimens from individuals with normal healthy conjunctivae without any clinical signs of trachoma (31 *vs* 23).

**Table 4:** Demographic details and the distribution of the severity of clinical signs of trachoma in the study and evaluation sample population

	All (Time-point 14)	Retained in test evaluation	Reference test		DjinniChip test	
			Ct-negative	Ct-positive	Ct-negative	Ct-positive
*N*	428	350	297	53	298	52
Median age (range)	10 (7–15)	10 (7–15)	10 (7–14)	9 (7–14)	10 (7–14)	9 (7–14)
Sex: female, *n* (%)	194 (45.3)	161 (46)	152 (52.9)	32 (60)	157 (52.7)	32 (61.5)
F-score, *n* (%)						
0	353 (82.5)	283 (80.9)	266 (89.6)	17 (32.1)	257 (86.2)	26 (50.0)
1	36 (8.4)	29 (8.3)	21 (7.1)	8 (15.1)	22 (7.4)	7 (13.5)
2	21 (4.9)	20 (5.7)	9 (3.0)	11 (20.7)	12 (4.0)	8 (15.4)
3	18 (4.2)	18 (5.1)	1 (0.3)	17 (32.1)	7 (2.4)	11 (21.2)
P-score, *n* (%)						
0	391 (91.3)	317 (90.6)	284 (95.6)	33 (62.2)	277 (93.0)	40 (76.9)
1	23 (5.4)	20 (5.7)	8 (2.7)	12 (22.6)	14 (4.7)	6 (11.5)
2	8 (1.9)	7 (2.0)	3 (1.0)	4 (7.6)	3 (1.0)	4 (7.7)
3	6 (1.4)	6 (1.7)	2 (0.7)	4 (7.6)	4 (1.3)	2 (3.9)
C-score, *n* (%)						
0	397 (92.8)	328 (93.7)	275 (92.9)	52 (98.1)	278 (93.6)	49 (94.2)
1	26 (6.0)	19 (5.4)	19 (6.4)	0	17 (5.7)	2 (3.9)
2	5 (1.2)	3 (0.9)	2 (0.7)	1 (1.9)	2 (0.7)	1 (1.9)
3	0	0	0	0	0	0

FPC-scores: Clinical signs were graded using the 1981 WHO Detailed Trachoma Grading System (FPC). This divides the clinical signs into several four-point (0–3) increasing severity scales: follicles (F), papillary inflammation (P), and conjunctival scarring (C). This system corresponds to the WHO Simplified Trachoma Grading System in the following way: trachomatous inflammation-follicular (TF) is equivalent to F2/F3 and trachomatous inflammation-intense (TI) is equivalent to P3. DjinniChip and qPCR testing was performed at KCRI-BL, Moshi, Tanzania

**Table 5:** Diagnostic performance of the DjinniChip on archived clinical samples

	qPCR-negative	qPCR-positive	Total
DjinniChip-negative	282^a^	18	300
DjinniChip-positive	17	35	52
Total	299	53	352

DjinniChip and qPCR testing were performed at KCRI-BL, Moshi, Tanzania

^a^Includes 2 air controls which tested negative by both qPCR and DjinniChip

**Table 6:** Diagnostic performance of the DjinniChip on archived clinical samples

	DjinniChip *vs* non-commercial qPCR	
	Value (%)	95% CI
Sensitivity	66.0	51.7–78.5
Specificity	94.3	91.1–96.7
PPV	67.3	55.5–77.3
NPV	94.0	91.5–95.8
Accuracy	90.1	86.4–93.0

DjinniChip and qPCR testing were performed at KCRI-BL, Moshi, Tanzania

### Field evaluation

In order to test the performance and usability of the DjinniChip in field conditions, a series of mock swabs spiked with *C. trachomatis* were tested at four field sites in northern Tanzania. Replicate mock swabs were also tested in the laboratory at KCRI-BL, Tanzania, for comparison.

In the laboratory, 8/8 *C. trachomatis*-positive mock swabs, ranging in concentration from 160–16,000 copies per swab (equivalent to 10–1000 copies per DjinniChip reaction), were correctly diagnosed as positive by the DjinniChip and by qPCR (Additional file 1: Table S2.). Duplicate negative control swabs were correctly diagnosed as negative by both methods.

In the field, 62/64 *C. trachomatis*-positive mock samples were correctly diagnosed as positive by the DjinniChip (Additional file 1: Table S3). There were two false negatives, both with concentrations of 50 copies per DjinniChip reaction; one tested by an experienced technician and one by a field nurse. In contrast, 10/16 negative control samples tested in the field were incorrectly diagnosed as *C. trachomatis* positive by the DjinniChip. Six false positives were tested by a field nurse and four by an experienced technician. qPCR testing confirmed that all 16 controls were *C. trachomatis* negative, suggesting that cross-contamination had occurred in the field after the sample lysis step. In the field, the time taken to test 10 samples was approximately 70 min (ranging from 135–150 min for 20 samples).

## Discussion

We developed and evaluated a LAMP-based first generation DjinniChip assay using ocular swabs collected in the course of a longitudinal trachoma cohort study in Moshi, Tanzania. DjinniChips enabled fast, simple processing and molecular diagnosis of *C. trachomatis* infection in resource limited environments typical of trachoma-endemic regions. The results demonstrated high specificity of the test (94.3%) but modest sensitivity (66%), which requires improvement before the DjinniChip can be considered for use in trachoma control programmes.

Sensitivity was affected by both false-positive results (17/52 (32.6%)) strongly impacting PPV, and false-negative results (18/300 (6%)) impacting NPV. The lower sensitivity of Djinnichip also resulted in a lower frequency of detection of infection in those with clinical signs of active trachoma but a higher frequency of infection in those with no clinical signs of disease. The results overall are more typical of an untreated trachoma-endemic population where current infection is detected in around 60% of active trachoma cases [15]. Indeed, the national trachoma control programme delivered a fourth round of community treatment with azithromycin in September 2015 to one of the three villages in this study where the prevalence of TF_1–9_ was > 10% as per the WHO recommendation.

The majority of the false-negative results were due to inhibition of the reaction, which could be overcome by dilution of the sample lysate. The assay design currently uses 25 µl of sample lysate (combined with 25 µl buffer); this large amount of sample lysate may negatively affect the performance of the test due to the presence of excess proteins (such as DNAses and proteases that are incompletely inactivated by heating), blood, inflammatory exudate, and dust. In 10 of 18 false-negative samples the inhibition was overcome by repeating the test with a 1:10 dilution of sample lysate to diluent. This is a well-described characteristic of NAATs which is overcome in the same manner with some commercial tests such as Roche CT/NG Amplicor [16]. Alternatively, stabilizing additives such as increased concentrations of albumin or sheared carrier DNA can be added to the diluent. Further sample dilution and the use of additives will be optimised in the next iteration of the DjinniChip.

The extreme sensitivity of LAMP to contaminating DNA most likely explains the false positivity rate [17–19]. DNA ‘contamination’ may occur either during DjinniChip manufacture or more likely during use. Standard precautions to avoid DNA contamination during manufacture and assembly were taken. The false-positive results observed were predominantly found in cases where the test result was ambiguous, evidenced by a faint band on the lateral flow strip. With the exception of one sample (with a high copy number), true positive samples were characterized by clear distinct bands, even when only a low copy number was present. DNA contamination during testing most likely occurred during sample lysis and loading, or post-amplification when the seal was removed to enable the amplified reaction mix to be pumped towards the lateral flow test strip. DNA contamination is frequently caused by aerosol and in the molecular laboratory this can be more easily controlled with aerosol barrier tips and the spatial/physical separation of the procedures. This is more difficult to control in a general laboratory, local health facility and under differing environmental conditions in the field where a closed system would offer a solution.

Although the susceptibility to sample inhibition and cross-contamination negatively affected the sensitivity of the test, other characteristics of the DjinniChip favour its use for rapid diagnostics in resource-limited settings. Due to the simplicity of the test, the DjinniChip can be used in the absence of laboratory infrastructure. The isothermal DNA amplification was performed on a simple and cheap 12V heating block powered from a car battery; its use requires minimal operator training and needs no software. All reagents are pre-stored on the chip and it requires storage at only 2–8 °C. Furthermore, sample preparation is relatively simple, and a test can be completed in 50 min in laboratory conditions, with assessment of test result by visual inspection. Other published isothermal assays for *C. trachomatis* are based on conventional laboratory processing and thus far have not been translated into suitable formats for use in low-resource settings [20–22]. Although there are some rapid and point-of-care tests available for *C. trachomatis* detection, there is currently no integrated molecular test developed for trachoma that is characterized by the same level of technical simplicity as the DjinniChip. Shin et al. [23] developed a mobile magnetofluidic platform for the extraction and amplification of *C. trachomatis* DNA (primarily designed for sexually transmitted infections); however, their device is reliant on a specialised instrument. Most recently, SelfDiagnostics Deutschland GmbH (Leipzig, Germany) released a fully integrated device for the detection of *C. trachomatis* and *N. gonorrehea* in urine (STD Multitest); the device is self-contained and suitable for home-use; however, its format (internal complexity and construction) results in high production costs making it suboptimal for neglected tropical disease control programmes [24].

The DjinniChip maintained stable performance over the entire duration of the study (> 4 weeks including shipping with storage at 4 °C). A preliminary accelerated ageing study performed by Fraunhofer IZI (data not shown) found that DjinniChip performance remained unaffected for at least 10 weeks when stored at ambient temperature (~ 23 °C). Field testing in Tanzania revealed that DjinniChip detection of *C. trachomatis* was not impeded by temperature ranges between 25–36 °C with humidity between 19–50%, even in two field sites with very dusty and windy conditions. The main factor affecting DjinniChip performance in the field was the high number of false positives, probably due to cross-contamination. In the field it was not possible to segregate each stage of sample preparation, DNA amplification and detection, as all processes were conducted in the open boot of a Land Rover, and over half of No Template Control samples were positive. Work towards the next iteration of DjinniChip therefore needs to focus on its translation into a fully closed integrated format with no manual handling of liquids in order to improve test specificity. DjinniChip sensitivity will also be improved by further investigation reagent thermostability and storage as well as the modifications that enable sample matrix to prevent inhibition. Despite the low cost and simple testing procedure (particularly in a fully closed integrated format), national programmes frequently visit > 1000 individuals during surveys and in a low prevalence setting it may be desirable to pool samples for infection testing [25]. In the next iteration of DjinniChip, the potential to increase sample throughput by pooling clinical samples needs to be assessed.

## Conclusions

The DjinniChip rapid molecular diagnostic test for *C. trachomatis* has promising potential; however, it requires further development to improve its suitability for use by trachoma control programmes. Its strengths are the simple operation, fast time-to-result, lack of requirements for proprietary or high-cost laboratory equipment, and its stability in challenging environmental conditions. The estimated approximate cost per test of < US$2 after scale-up makes it a suitable candidate for diagnostic programmes in the future. The current limitations of the DjinniChip are its susceptibility to DNA contamination due to the manual sample- and detection buffer-handling steps and sporadic inhibition of the reaction by sample matrix, both of which can be eliminated in the next iteration. We were also limited by our reduced sample size due to sample loss and higher than expected prevalence of ocular infection. For the DjinniChip to be useful for trachoma control programmatic decisions post-MDA only a modest improvement in sensitivity may be required to maintain sensitivity of the test at lower infection prevalence associated with elimination targets. Conversely, the higher than expected infection prevalence with the reduced sample size may have inflated the performance of the index test specificity. The test, most likely, currently has sufficient high specificity since it would be used to make treatment decisions at the community level. The DjinniChip was designed for use by non-specialists in district level laboratories or health centres in trachoma endemic areas for the surveillance of *C. trachomatis*. Following improvements identified above, the DjinniChip holds promising potential to prevent unnecessary rounds of MDA and to rapidly detect recrudescence of infection in the community.

## Supplementary information

** Additional file 1: Table S1. **STARD checklist.** Table S2. **Raw results of qPCR C. trachomatis NAATs v DjinniChip.** Table S3.** Results of the field evaluation of DjinniChips with mock swabs. The DjinniChip tests were performed by an experienced user (Exp.) and a lay user. qPCR was performed by a qualified operator using swab material eluted in the field by experienced and lay users.** Figure S1.** Example results of lateral flow detection: a, positive result; b, negative result; c and d, undetermined results (weakly positive bands, indicated with arrows), require confirmation.** Figure S2.** Preliminary evaluation of DjinniChips with clinical samples. Four hundred microliters of elution buffer was added to each swab. Two hundred µl was used for DNA extraction with QIAamp DNA mini kit, eluted in 100 µl and tested by ddPCR. The remaining eluate was heat-lysed for 10 min at 95 °C; 25 µl of the lysate was mixed with 25 µl reaction buffer (20 mM Tris-HCl, 10 mM (NH4)2SO4, 150 mM KCl, 2 mM MgSO4, 0.1% Tween® 20, 1% Triton X-100, pH 8.8) and loaded onto the DjinniChip. LAMP was performed for 35 min at 65 °C on a flatbed heating block. **Figure S3.** DjinniChip results of Quality Control for Molecular Diagnostics DNA samples. A QCMD-panel of C. trachomatis DNA samples was obtained from the External Quality Assessment programme (https://www.qcmd.org; Glasgow, UK). The QCMD panel is a set of purified DNA samples from different sources and various concentrations. The samples were stored at -20 °C prior testing. Ten microliters of each QCMD sample were mixed with 40 µl reaction buffer and loaded onto the DjinniChip. LAMP was performed for 35 min at 65 °C on a flatbed heating block.

## Data Availability

Data cannot be shared publicly without a request for a data transfer agreement from the Tanzania national ethics committee. Individual requests for transfer of data can be directed to National Institute for Medical Research in Tanzania (contact *via* ethics@nimr.or. tz) for researchers who meet the criteria for access to confidential data.

## Abbreviations

omcB: Outer membrane complex B
ORF: Open reading frame
LAMP: Loop-mediated isothermal amplification
LFS: Lateral flow strip
MDA: Mass drug administration
NAAT: Nucleic acid amplification test
NPV: Negative predictive value
PPV: Positive predictive value
QCMD: Quality control for molecular diagnostics
qPCR: Quantitative polymerase chain reaction
RPP30: Ribonuclease P/MRP Subunit P30
SAFE: Surgery for trichiasis (in-turned eyelashes), Antibiotics, Facial cleanliness, and Environmental improvement
TF: Trachomatous inflammation-follicular
WHO: World Health Organisation

## References

[1] Mariotti SP, Pascolini D, Rose-Nussbaumer J (2009). Trachoma: global magnitude of a preventable cause of blindness. Br J Ophthalmol..

[2] Ramadhani AM, Macleod D, Holland MJ, Burton MJ, Derrick T (2016). The relationship between active trachoma and ocular *Chlamydia trachomatis* infection before and after mass antibiotic treatment. PLoS Negl Trop Dis..

[3] PATH. Target Product Profile: Trachoma surveillance diagnostic–antigen lateral flow test; 2015. https://www.path.org/resources/tpp-trachoma-antigen-lateral-flow-test/.

[4] Solomon AW, Engels D, Bailey RL, Blake IM, Brooker S, Chen J-X (2012). A diagnostics platform for the integrated mapping, monitoring, and surveillance of neglected tropical diseases: rationale and target product profiles. PLoS Negl Trop Dis..

[5] Harding-Esch EM, Cousins EC, Chow SC, Phillips LT, Hall CL, Cooper N (2018). A 30-min nucleic acid amplification point-of-care test for genital *Chlamydia trachomatis* infection in women: a prospective, multi-center study of diagnostic accuracy. EBioMed.

[6] Kelly H, Coltart CEM, Pant Pai N, Klausner JD, Unemo M, Toskin I (2017). Systematic reviews of point-of-care tests for the diagnosis of urogenital *Chlamydia trachomatis* infections. Sex Transm Infect..

[7] Ramadhani AM, Derrick T, Macleod D, Massae P, Mtuy T, Jeffries D (2017). Immunofibrogenic gene expression patterns in Tanzanian children with ocular *Chlamydia trachomatis* infection, active trachoma and scarring: baseline results of a 4-year longitudinal study. Front Cell Infect Microbiol..

[8] Ramadhani AM, Derrick T, Macleod D, Massae P, Malisa A, Mbuya K (2019). Ocular immune responses, *Chlamydia trachomatis* infection and clinical signs of trachoma before and after azithromycin mass drug administration in a treatment naïve trachoma-endemic Tanzanian community. PLoS Negl Trop Dis..

[9] Ramadhani AM, Derrick T, MacLeod D, Massae P, Mafuru E, Malisa A (2019). Progression of scarring trachoma in Tanzanian children: a 4-year cohort study. PLoS Negl Trop Dis..

[10] Butcher R, Houghton J, Derrick T, Ramadhani A, Herrera B, Last AR (2017). Reduced-cost *Chlamydia trachomatis*-specific multiplex real-time PCR diagnostic assay evaluated for ocular swabs and use by trachoma research programmes. J Microbiol Methods..

[11] Faal N, Bailey RL, Jeffries D, Joof H, Sarr I, Laye M (2006). Conjunctival FOXP3 expression in trachoma: Do regulatory T cells have a role in human ocular *Chlamydia trachomatis* infection?. PLoS Med..

[12] Bossuyt PM, Reitsma JB, Bruns DE, Gatsonis CA, Glasziou PP, Irwig L (2015). STARD 2015: an updated list of essential items for reporting diagnostic accuracy studies. Clin Chem..

[13] Derrick T, Last AR, Burr SE, Roberts CH, Nabicassa M, Cassama E (2016). Inverse relationship between microRNA-155 and -184 expression with increasing conjunctival inflammation during ocular *Chlamydia trachomatis* infection. BMC Infect Dis..

[14] Pickering H, Teng A, Faal N, Joof H, Makalo P, Cassama E (2017). Genome-wide profiling of humoral immunity and pathogen genes under selection identifies immune evasion tactics of *Chlamydia trachomatis* during ocular infection. Sci Rep..

[15] Last AR, Burr SE, Weiss HA, Harding-Esch EM, Cassama E, Nabicassa M (2014). Risk factors for active trachoma and ocular *Chlamydia trachomatis* infection in treatment-naïve trachoma-hyperendemic communities of the Bijagós Archipelago, Guinea Bissau. PLoS Negl Trop Dis.

[16] Harding-Esch EM, Holland MJ, Schémann J-F, Molina S, Sarr I, Andreasen AA (2011). Diagnostic accuracy of a prototype point-of-care test for ocular *Chlamydia trachomatis* under field conditions in the Gambia and Senegal. PLoS Negl Trop Dis..

[17] Zhou D, Guo J, Xu L, Gao S, Lin Q, Wu Q (2014). Establishment and application of a loop-mediated isothermal amplification (LAMP) system for detection of cry1Ac transgenic sugarcane. Sci Rep..

[18] Karthik K, Rathore R, Thomas P, Arun TR, Viswas KN, Dhama K (2014). New closed tube loop mediated isothermal amplification assay for prevention of product cross-contamination. MethodsX..

[19] Ma C, Wang F, Wang X, Han L, Jing H, Zhang H (2017). A novel method to control carryover contamination in isothermal nucleic acid amplification. Chem Commun..

[20] Choopara I, Arunrut N, Kiatpathomchai W, Dean D, Somboonna N (2017). Rapid and visual *Chlamydia trachomatis* detection using loop-mediated isothermal amplification and hydroxynaphthol blue. Lett Appl Microbiol..

[21] Krolov K, Frolova J, Tudoran O, Suhorutsenko J, Lehto T, Sibul H (2014). Sensitive and rapid detection of *Chlamydia trachomatis* by recombinase polymerase amplification directly from urine samples. J Mol Diagn..

[22] Crotchfelt KA, Pare B, Gaydos C, Quinn TC (1998). Detection of *Chlamydia trachomatis* by the Gen-Probe AMPLIFIED Chlamydia Trachomatis Assay (AMP CT) in urine specimens from men and women and endocervical specimens from women. J Clin Microbiol..

[23] Shin DJ, Athamanolap P, Chen L, Hardick J, Lewis M, Hsieh YH (2017). Mobile nucleic acid amplification testing (mobiNAAT) for *Chlamydia trachomatis* screening in hospital emergency department settings. Sci Rep..

[24] Selfdiagnostics. STD MULTITEST; 2019. https://selfdiagnostics.eu/std-multitest/.

[25] Dize L, West S, Quinn TC, Gaydos CA (2014). Pooling ocular swab specimens from Tanzania for testing by Roche Amplicor and Aptima Combo 2 assays for the detection of *Chlamydia trachomatis*: accuracy and Cost Savings. Diagn Microbiol Infect Dis..

