# Supplementary material for: DjinniChip: evaluation of a novel molecular rapid diagnostic device for the detection of Chlamydia trachomatis in trachoma-endemic areas
Source: Parasit Vectors. 2020 Oct 27;13:533. doi: 10.1186/s13071-020-04414-6 (PMC7590679; doi:10.1186/s13071-020-04414-6)
Supplement: Supplementary file 1 — Additional file 1: Table S1. STARD checklist. Table S2. Raw results of qPCR C. trachomatis NAATs v DjinniChip. Table S3. Results of the field evaluation of DjinniChips with mock swabs. The DjinniChip tests were performed by an experienced user (Exp.) and a lay user. qPCR was performed by a qualified operator using swab material eluted in the field by experienced and lay users. Figure S1. Example results of lateral flow detection: a, positive result; b, negative result; c and d, undetermined results (weakly positive bands, indicated with arrows), require confirmation. Figure S2. Preliminary evaluation of DjinniChips with clinical samples. Four hundred microliters of elution buffer was added to each swab. Two hundred µl was used for DNA extraction with QIAamp DNA mini kit, eluted in 100 µl and tested by ddPCR. The remaining eluate was heat-lysed for 10 min at 95 °C; 25 µl of the lysate was mixed with 25 µl reaction buffer (20 mM Tris-HCl, 10 mM (NH4)2SO4, 150 mM KCl, 2 mM MgSO4, 0.1% Tween® 20, 1% Triton X-100, pH 8.8) and loaded onto the DjinniChip. LAMP was performed for 35 min at 65 °C on a flatbed heating block. Figure S3. DjinniChip results of Quality Control for Molecular Diagnostics DNA samples. A QCMD-panel of C. trachomatis DNA samples was obtained from the External Quality Assessment programme (https://www.qcmd.org; Glasgow, UK). The QCMD panel is a set of purified DNA samples from different sources and various concentrations. The samples were stored at -20 °C prior testing. Ten microliters of each QCMD sample were mixed with 40 µl reaction buffer and loaded onto the DjinniChip. LAMP was performed for 35 min at 65 °C on a flatbed heating block. [file 13071_2020_4414_MOESM1_ESM.docx]

**Additional file 1**

**Figure S1.** Example results of lateral flow detection. a: positive result, b: negative result, c and d: undetermined results (weakly positive bands, indicated with arrows), require confirmation.

.


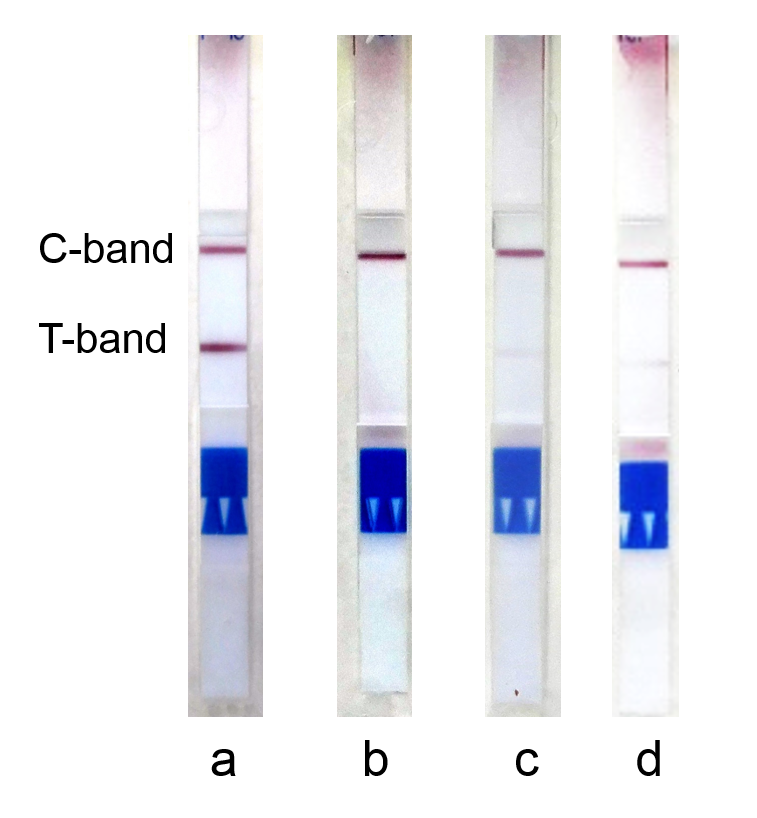


**Figure S2.** Preliminary evaluation of DjinniChips with clinical samples. Four hundred microliters of elution buffer was added to each swab. Two hundred µl was used for DNA extraction with QIAamp DNA mini kit, eluted in 100 µl and tested by ddPCR. The remaining swab eluate was heat-lysed for 10 min at 95°C; 25 µl of the lysate was mixed with 25 µl reaction buffer (20 mM Tris-HCl, 10 mM (NH_4_)_2_SO_4_, 150 mM KCl, 2 mM MgSO_4_, 0.1% Tween® 20, 1% Triton X-100, pH 8.8) and loaded onto the chip. LAMP was performed for 35 min at 65°C on a flatbed heating block.

**
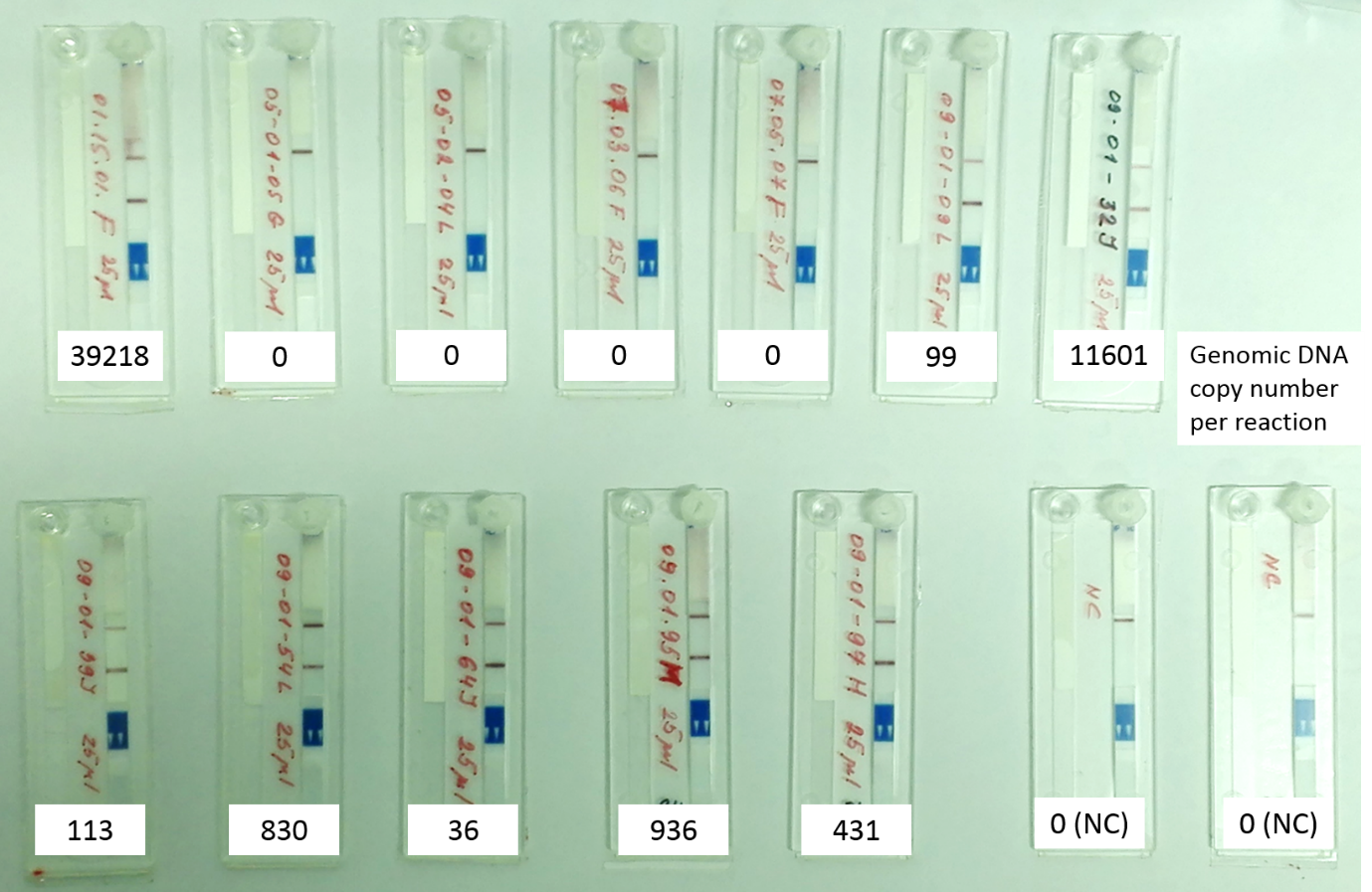
**

**Figure S3.** Preliminary evaluation of DjinniChips with QCMD panel. A Quality Control for Molecular Diagnostics (QCMD)-panel of *Chlamydia trachomatis* DNA samples was obtained via External Quality Assessment programme (https://www.qcmd.org; Glasgow, UK). A QCMD panel is a set of purified DNA samples from different sources and of various concentrations. The samples were stored at -20°C prior testing. Ten microliters of each QCMD sample were mixed with 40 µl reaction buffer and loaded onto the DjinniChip. LAMP was performed for 35 min at 65°C on a flatbed heating block.


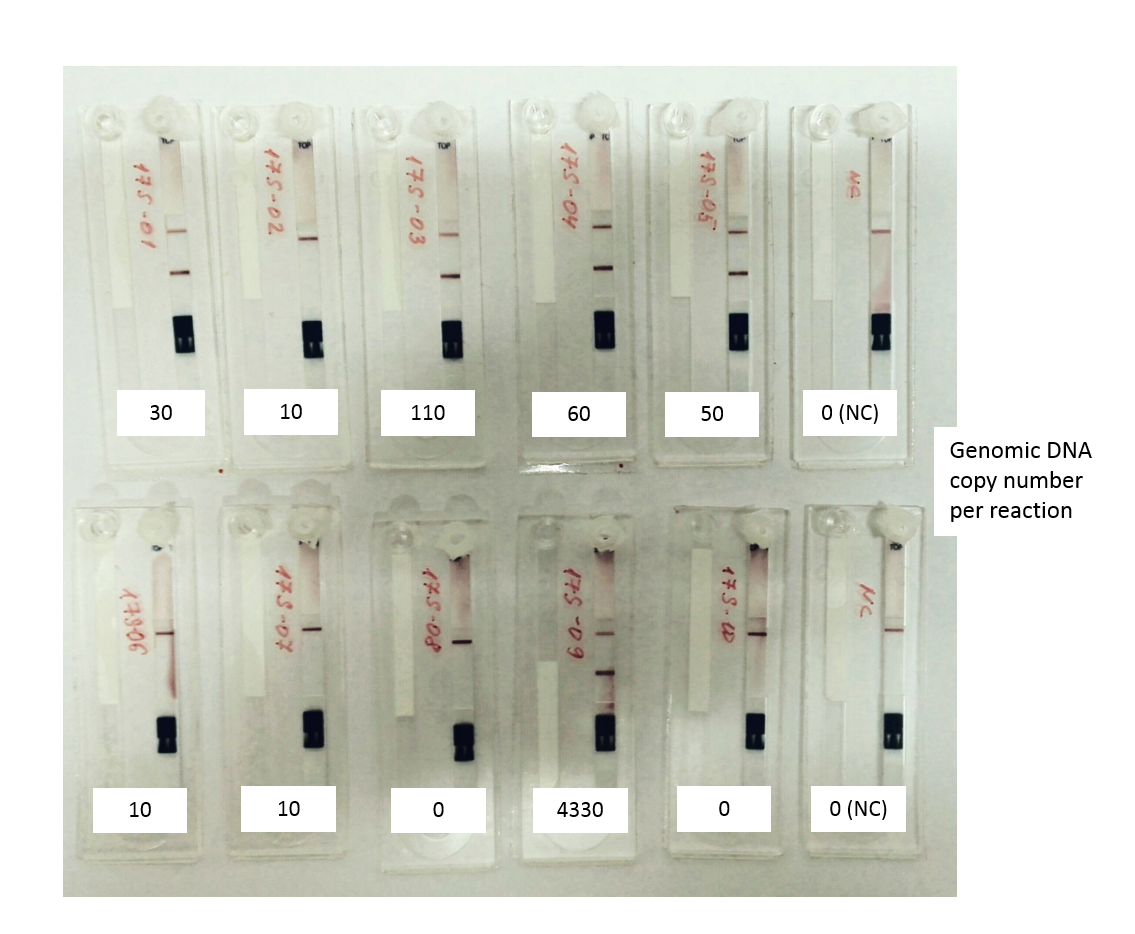


**Table S1**. STARD 2015 Checklist

|  | **Section & Topic** | **No** | **Item** | **Reported on page #** |
| --- | --- | --- | --- | --- |
|  |  |  |  |  |
|  | **TITLE OR ABSTRACT** |  |  |  |
|  |  | **1** | Identification as a study of diagnostic accuracy using at least one measure of accuracy  (such as sensitivity, specificity, predictive values, or AUC) | P2 |
|  | **ABSTRACT** |  |  |  |
|  |  | **2** | Structured summary of study design, methods, results, and conclusions  (for specific guidance, see STARD for Abstracts) | P2 |
|  | **INTRODUCTION** |  |  |  |
|  |  | **3** | Scientific and clinical background, including the intended use and clinical role of the index test | P3 |
|  |  | **4** | Study objectives and hypotheses | P3 |
|  | **METHODS** |  |  |  |
|  | *Study design* | **5** | Whether data collection was planned before the index test and reference standard were performed (prospective study) or after (retrospective study) | P4 |
|  | *Participants* | **6** | Eligibility criteria | P4 |
|  |  | **7** | On what basis potentially eligible participants were identified  (such as symptoms, results from previous tests, inclusion in registry) | P4 |
|  |  | **8** | Where and when potentially eligible participants were identified (setting, location and dates) | P4 |
|  |  | **9** | Whether participants formed a consecutive, random or convenience series | P4 |
|  | *Test methods* | **10a** | Index test, in sufficient detail to allow replication | P5 |
|  |  | **10b** | Reference standard, in sufficient detail to allow replication | P4 |
|  |  | **11** | Rationale for choosing the reference standard (if alternatives exist) | P4 |
|  |  | **12a** | Definition of and rationale for test positivity cut-offs or result categories  of the index test, distinguishing pre-specified from exploratory | P6 |
|  |  | **12b** | Definition of and rationale for test positivity cut-offs or result categories  of the reference standard, distinguishing pre-specified from exploratory | P4 |
|  |  | **13a** | Whether clinical information and reference standard results were available  to the performers/readers of the index test | P6 |
|  |  | **13b** | Whether clinical information and index test results were available  to the assessors of the reference standard | P6 |
|  | *Analysis* | **14** | Methods for estimating or comparing measures of diagnostic accuracy | TABLE 5 |
|  |  | **15** | How indeterminate index test or reference standard results were handled | P7 |
|  |  | **16** | How missing data on the index test and reference standard were handled | - |
|  |  | **17** | Any analyses of variability in diagnostic accuracy, distinguishing pre-specified from exploratory | - |
|  |  | **18** | Intended sample size and how it was determined | P4 |
|  | **RESULTS** |  |  |  |
|  | *Participants* | **19** | Flow of participants, using a diagram | - (P9) |
|  |  | **20** | Baseline demographic and clinical characteristics of participants | TABLE 4 |
|  |  | **21a** | Distribution of severity of disease in those with the target condition | TABLE 4 |
|  |  | **21b** | Distribution of alternative diagnoses in those without the target condition | - |
|  |  | **22** | Time interval and any clinical interventions between index test and reference standard | P5 |
|  | *Test results* | **23** | Cross tabulation of the index test results (or their distribution)  by the results of the reference standard | TABLE 5 |
|  |  | **24** | Estimates of diagnostic accuracy and their precision (such as 95% confidence intervals) | TABLE 6 |
|  |  | **25** | Any adverse events from performing the index test or the reference standard | - |
|  | **DISCUSSION** |  |  |  |
|  |  | **26** | Study limitations, including sources of potential bias, statistical uncertainty, and generalisability | P12 |
|  |  | **27** | Implications for practice, including the intended use and clinical role of the index test | P13 |
|  | **OTHER INFORMATION** |  |  |  |
|  |  | **28** | Registration number and name of registry | - |
|  |  | **29** | Where the full study protocol can be accessed | - |
|  |  | **30** | Sources of funding and other support; role of funders | P15 |
|  |  |  |  |  |

**Table S2.** Per sample results of qPCR and DjinniChip assay

| Raw results of qPCR *Chlamydia trachomatis* *vs* DjinniChip | | | | | | |
| --- | --- | --- | --- | --- | --- | --- |
| Sample ID | DjinniChip | | PCR | Chromosome target, copies/µl | Plasmid target, copies/µl | Post-PCR confirmatory test |
| CD14-001 | neg | neg | | 0 | 0 |  |
| CD14-002 | neg | neg | | 0 | 0 |  |
| CD14-003 | neg | neg | | 0 | 0 |  |
| CD14-004 | neg | neg | | 0 | 0 |  |
| CD14-005 | neg | neg | | 0 | 0 |  |
| CD14-006 | neg | neg | | 0 | 0 |  |
| CD14-007 | neg | neg | | 0 | 0 |  |
| CD14-008 | neg | neg | | 0 | 0 |  |
| CD14-009 | neg | neg | | 0 | 0 |  |
| CD14-010 | neg | neg | | 0 | 0 |  |
| CD14-011 | neg | neg | | 0 | 0 |  |
| CD14-012 | neg | neg | | 0 | 0 |  |
| CD14-013 | neg | neg | | 0 | 0 |  |
| CD14-014 | neg | neg | | 0 | 0 |  |
| CD14-015 | neg | neg | | 0 | 0 |  |
| CD14-016 | neg | neg | | 0 | 0 |  |
| CD14-017 | neg | neg | | 0 | 0 |  |
| CD14-018 | neg | neg | | 0 | 0 |  |
| CD14-019 | neg | neg | | 0 | 0 |  |
| CD14-020 | neg | neg | | 0 | 0 |  |
| CD14-021 | pos | neg | | 0 | 0 |  |
| CD14-022 | neg | neg | | 0 | 0 |  |
| CD14-023 | neg | neg | | 0 | 0 |  |
| CD14-024 | neg | neg | | 0 | 0 |  |
| CD14-025 | neg | neg | | 0 | 0 |  |
| CD14-026 | neg | neg | | 0 | 0 |  |
| CD14-027 | neg | neg | | 0 | 0 |  |
| CD14-028 | pos | neg | | 0 | 0 |  |
| CD14-029 | neg | neg | | 0 | 0 |  |
| CD14-030 | neg | neg | | 0 | 0 |  |
| CD14-031 | neg | neg | | 0 | 0 |  |
| CD14-032 | neg | neg | | 0 | 0 |  |
| CD14-033 | neg | neg | | 0 | 0 |  |
| CD14-034 | neg | neg | | 0 | 0 |  |
| CD14-035 | neg | neg | | 0 | 0 |  |
| CD14-036 | neg | neg | | 0 | 0 |  |
| CD14-037 | neg | neg | | 0 | 0 |  |
| CD14-038 | neg | neg | | 0 | 0 |  |
| CD14-039 | neg | neg | | 0 | 0 |  |
| CD14-040 | neg | neg | | 0 | 0 |  |
| CD14-041 | neg | neg | | 0 | 0 |  |
| CD14-042 | neg | neg | | 0 | 0 |  |
| CD14-043 | neg | neg | | 0 | 0 |  |
| CD14-044 | neg | neg | | 0 | 0 |  |
| CD14-045 | neg | neg | | 0 | 0 |  |
| CD14-046 | neg | neg | | 0 | 0 |  |
| CD14-047 | neg | neg | | 0 | 0 |  |
| CD14-048 | neg | neg | | 0 | 0 |  |
| CD14-049 | neg | neg | | 0 | 0 |  |
| CD14-050 | neg | neg | | 0 | 0 |  |
| CD14-051 | neg | neg | | 0 | 0 |  |
| CD14-052 | neg | neg | | 0 | 0 |  |
| CD14-053 | neg | neg | | 0 | 0 |  |
| CD14-054 | neg | neg | | 0 | 0 |  |
| CD14-055 | neg | neg | | 0 | 0 |  |
| CD14-056 | neg | neg | | 0 | 0 |  |
| CD14-057 | neg | neg | | 0 | 0 |  |
| CD14-058 | neg | neg | | 0 | 0 |  |
| CD14-059 | neg | neg | | 0 | 0 |  |
| CD14-060 | neg | neg | | 0 | 0 |  |
| CD14-061 | neg | neg | | 0 | 0 |  |
| CD14-062 | neg | neg | | 0 | 0 |  |
| CD14-063 | neg | neg | | 0 | 0 |  |
| CD14-064 | neg | neg | | 0 | 0 |  |
| CD14-065 | neg | neg | | 0 | 0 |  |
| CD14-066 | neg | neg | | 0 | 0 |  |
| CD14-067 | neg | neg | | 0 | 0 |  |
| CD14-068 | neg | neg | | 0 | 0 |  |
| CD14-069 | neg | neg | | 0 | 0 |  |
| CD14-070 | pos | pos | | 150803.0 | 332147.8 |  |
| CD14-071 | neg | neg | | 0 | 0 |  |
| CD14-072 | neg | neg | | 0 | 0 |  |
| CD14-073 | neg | neg | | 0 | 0 |  |
| CD14-074 | neg | neg | | 0 | 0 |  |
| CD14-075 | neg | neg | | 0 | 0 |  |
| CD14-076 | neg | neg | | 0 | 0 |  |
| CD14-077 | neg | neg | | 0 | 0 |  |
| CD14-078 | neg | neg | | 0 | 0 |  |
| CD14-079 | neg | neg | | 0 | 0 |  |
| CD14-080 | neg | neg | | 0 | 0 |  |
| CD14-081 | neg | neg | | 0 | 0 |  |
| CD14-082 | neg | neg | | 0 | 0 |  |
| CD14-083 | neg | neg | | 0 | 0 |  |
| CD14-084 | neg | neg | | 0 | 0 |  |
| CD14-085 | neg | neg | | 0 | 0 |  |
| CD14-086 | neg | neg | | 0 | 0 |  |
| CD14-087 | neg | neg | | 0 | 0 |  |
| CD14-088 | neg | neg | | 0 | 0 |  |
| CD14-089 | neg | neg | | 0 | 0 |  |
| CD14-090 | neg | neg | | 0 | 0 |  |
| CD14-091 | neg | neg | | 0 | 0 |  |
| CD14-092 | neg | neg | | 0 | 0 |  |
| CD14-093 | neg | neg | | 0 | 0 |  |
| CD14-094 | neg | neg | | 0 | 0 |  |
| CD14-095 | neg | neg | | 0 | 0 |  |
| CD14-096 | neg | neg | | 0 | 0 |  |
| CD14-097 | neg | neg | | 0 | 0 |  |
| CD14-098 | neg | neg | | 0 | 0 |  |
| CD14-099 | neg | neg | | 0 | 0 |  |
| CD14-100 | neg | neg | | 0 | 0 |  |
| CD14-179 | neg | neg | | 0 | 0 |  |
| CD14-180 | neg | neg | | 0 | 0 |  |
| CD14-181 | neg | neg | | 0 | 0 |  |
| CD14-182 | neg | neg | | 0 | 0 |  |
| CD14-183 | neg | neg | | 0 | 0 |  |
| CD14-184 | neg | neg | | 0 | 0 |  |
| CD14-185 | neg | neg | | 0 | 0 |  |
| CD14-186 | neg | neg | | 0 | 0 |  |
| CD14-187 | neg | neg | | 0 | 0 |  |
| CD14-188 | neg | neg | | 0 | 0 |  |
| CD14-189 | neg | neg | | 0 | 0 |  |
| CD14-190 | neg | neg | | 0 | 0 |  |
| CD14-191 | neg | neg | | 0 | 0 |  |
| CD14-192 | neg | neg | | 0 | 0 |  |
| CD14-193 | neg | neg | | 0 | 0 |  |
| CD14-194 | neg | neg | | 0 | 0 |  |
| CD14-195 | neg | neg | | 0 | 0 |  |
| CD14-196 | neg | neg | | 0 | 0 |  |
| CD14-197 | neg | neg | | 0 | 0 |  |
| CD14-198 | neg | neg | | 0 | 0 |  |
| CD14-199 | neg | neg | | 0 | 0 |  |
| CD14-200 | neg | neg | | 0 | 0 |  |
| CD14-201 | neg | neg | | 0 | 0 |  |
| CD14-202 | neg | neg | | 0 | 0 |  |
| CD14-203 | pos | pos | | 2236.7 | 6547.3 |  |
| CD14-204 | neg | neg | | 0 | 0 |  |
| CD14-205 | neg | neg | | 0 | 0 |  |
| CD14-206 | neg | neg | | 0 | 0 |  |
| CD14-207 | neg | neg | | 0 | 0 |  |
| CD14-208 | pos | pos | | 454.7 | 1398.6 |  |
| CD14-209 | neg | neg | | 0 | 0 |  |
| CD14-210 | neg | neg | | 0 | 0 |  |
| CD14-211 | neg | neg | | 0 | 0 |  |
| CD14-212 | neg | neg | | 0 | 0 |  |
| CD14-213 | neg | pos | | 0 | 10.9 | (1:10) neg |
| CD14-214 | neg | neg | | 0 | 0 |  |
| CD14-215 | neg | neg | | 0 | 0 |  |
| CD14-216 | neg | neg | | 0 | 0 |  |
| CD14-217 | neg | neg | | 0 | 0 |  |
| CD14-218 | neg | neg | | 0 | 0 |  |
| CD14-219 | neg | neg | | 0 | 0 |  |
| CD14-220 | neg | neg | | 0 | 0 |  |
| CD14-221 | neg | neg | | 0 | 0 |  |
| CD14-222 | neg | neg | | 0 | 0 |  |
| CD14-223 | neg | neg | | 0 | 0 |  |
| CD14-224 | neg | pos | | 1167.5 | 6093.8 | (1:10) pos |
| CD14-225 | neg | pos | | 1591.4 | 4794.5 | (1:10) pos |
| CD14-226 | neg | pos | | 36559.0 | 122852.9 | (1:10) pos |
| CD14-227 | neg | pos | | 3331.1 | 4001.2 | (1:10) pos |
| CD14-228 | neg | neg | | 0 | 0 |  |
| CD14-229 | neg | neg | | 0 | 0 |  |
| CD14-230 | neg | pos | | 32184.7 | 19211.9 | (1:10) pos |
| CD14-231 | neg | neg | | 0 | 0 |  |
| CD14-232 | pos | neg | | 0 | 0 |  |
| CD14-233 | pos | pos | | 2973.8 | 6069.0 |  |
| CD14-234 | neg | neg | | 0 | 0 |  |
| CD14-235 | pos | pos | | 10824.5 | 30371.8 |  |
| CD14-236 | neg | neg | | 0 | 0 |  |
| CD14-237 | neg | neg | | 0 | 0 |  |
| CD14-238 | neg | pos | | 1907.2 | 8125.8 | (1:10) pos |
| CD14-239 | neg | neg | | 0 | 0 |  |
| CD14-240 | neg | neg | | 0 | 0 |  |
| CD14-241 | neg | pos | | 77.9 | 464.4 | (1:10) pos |
| CD14-242 | neg | neg | | 0 | 0 |  |
| CD14-243 | neg | neg | | 0 | 0 |  |
| CD14-244 | neg | neg | | 0 | 0 |  |
| CD14-245 | neg | pos | | 245.9 | 697.2 | (1:10) pos |
| CD14-246 | neg | neg | | 0 | 0 |  |
| CD14-247 | neg | pos | | 3.7 | 0 | (NA) |
| CD14-248 | neg | neg | | 0 | 0 |  |
| CD14-249 | neg | pos | | 6873.3 | 22977.7 | (1:10) pos |
| CD14-250 | neg | neg | | 0 | 0 |  |
| CD14-251 | neg | neg | | 0 | 0 |  |
| CD14-252 | neg | neg | | 0 | 0 |  |
| CD14-253 | neg | neg | | 0 | 0 |  |
| CD14-254 | neg | neg | | 0 | 0 |  |
| CD14-255 | neg | neg | | 0 | 0 |  |
| CD14-256 | pos | pos | | 101.6 | 607.2 |  |
| CD14-257 | neg | neg | | 0 | 0 |  |
| CD14-258 | neg | neg | | 0 | 0 |  |
| CD14-259 | neg | neg | | 0 | 0 |  |
| CD14-260 | neg | neg | | 0 | 0 |  |
| CD14-261 | neg | neg | | 0 | 0 |  |
| CD14-262 | neg | neg | | 0 | 0 |  |
| CD14-263 | neg | neg | | 0 | 0 |  |
| CD14-264 | neg | neg | | 0 | 0 |  |
| CD14-265 | neg | neg | | 0 | 0 |  |
| CD14-266 | neg | neg | | 0 | 0 |  |
| CD14-267 | neg | neg | | 0 | 0 |  |
| CD14-268 | neg | neg | | 0 | 0 |  |
| CD14-269 | neg | neg | | 0 | 0 |  |
| CD14-270 | neg | neg | | 0 | 0 |  |
| CD14-271 | neg | neg | | 0 | 0 |  |
| CD14-272 | neg | neg | | 0 | 0 |  |
| CD14-273 | neg | neg | | 0 | 0 |  |
| CD14-274 | neg | neg | | 0 | 0 |  |
| CD14-275 | neg | neg | | 0 | 0 |  |
| CD14-276 | neg | neg | | 0 | 0 |  |
| CD14-277 | pos | pos | | 425.6 | 1506.0 |  |
| CD14-278 | neg | neg | | 0 | 0 |  |
| CD14-279 | pos | neg | | 0 | 0 |  |
| CD14-280 | neg | neg | | 0 | 0 |  |
| CD14-281 | neg | neg | | 0 | 0 |  |
| CD14-282 | neg | neg | | 0 | 0 |  |
| CD14-283 | neg | pos | | 6.8 | 0 | (NA) |
| CD14-284 | neg | neg | | 0 | 0 |  |
| CD14-285 | neg | neg | | 0 | 0 |  |
| CD14-286 | pos | pos | | 1239.6 | 3655.8 |  |
| CD14-287 | pos | neg | | 0 | 0 |  |
| CD14-288 | neg | neg | | 0 | 0 |  |
| CD14-289 | pos | neg | | 0 | 0 |  |
| CD14-290 | neg | neg | | 0 | 0 |  |
| CD14-291 | neg | neg | | 0 | 0 |  |
| CD14-292 | neg | neg | | 0 | 0 |  |
| CD14-293 | neg | neg | | 0 | 0 |  |
| CD14-294 | pos | pos | | 0 | 12.1 |  |
| CD14-295 | pos | pos | | 28.2 | 178.5 |  |
| CD14-296 | neg | neg | | 0 | 0 |  |
| CD14-297 | pos | pos | | 608.2 | 3874.0 |  |
| CD14-298 | neg | neg | | 0 | 0 |  |
| CD14-299 | pos | neg | | 0 | 0 |  |
| CD14-300 | neg | neg | | 0 | 0 |  |
| CD14-301 | pos | pos | | 226.7 | 475.8 |  |
| CD14-302 | neg | neg | | 0 | 0 |  |
| CD14-303 | pos | pos | | 295.9 | 891.7 |  |
| CD14-304 | pos | pos | | 5202.6 | 18112.4 |  |
| CD14-305 | pos | pos | | 414.5 | 2715.9 |  |
| CD14-306 | neg | neg | | 0 | 0 |  |
| CD14-307 | neg | neg | | 0 | 0 |  |
| CD14-308 | pos | pos | | 32.1 | 265.0 |  |
| CD14-309 | neg | neg | | 0 | 0 |  |
| CD14-310 | neg | neg | | 0 | 0 |  |
| CD14-311 | neg | neg | | 0 | 0 |  |
| CD14-312 | neg | pos | | 3.6 | 0 | (NA) |
| CD14-313 | neg | neg | | 0 | 0 |  |
| CD14-314 | neg | neg | | 0 | 0 |  |
| CD14-315 | neg | neg | | 0 | 0 |  |
| CD14-316 | neg | neg | | 0 | 0 |  |
| CD14-317 | neg | neg | | 0 | 0 |  |
| CD14-318 | neg | neg | | 0 | 0 |  |
| CD14-319 | neg | neg | | 0 | 0 |  |
| CD14-320 | neg | neg | | 0 | 0 |  |
| CD14-321 | pos | neg | | 0 | 0 |  |
| CD14-322 | neg | neg | | 0 | 0 |  |
| CD14-323 | pos | neg | | 0 | 0 |  |
| CD14-324 | neg | neg | | 0 | 0 |  |
| CD14-325 | pos | neg | | 0 | 0 |  |
| CD14-326 | pos | pos | | 2057.9 | 8462.7 |  |
| CD14-327 | pos | neg | | 0 | 0 |  |
| CD14-328 | neg | neg | | 0 | 0 |  |
| CD14-329 | neg | neg | | 0 | 0 |  |
| CD14-330 | neg | neg | | 0 | 0 |  |
| CD14-331 | neg | neg | | 0 | 0 |  |
| CD14-332 | neg | neg | | 0 | 0 |  |
| CD14-333 | neg | neg | | 0 | 0 |  |
| CD14-334 | neg | neg | | 0 | 0 |  |
| CD14-335 | neg | neg | | 0 | 0 |  |
| CD14-336 | neg | neg | | 0 | 0 |  |
| CD14-337 | neg | neg | | 0 | 0 |  |
| CD14-338 | neg | neg | | 0 | 0 |  |
| CD14-339 | pos | neg | | 0 | 0 |  |
| CD14-340 | neg | neg | | 0 | 0 |  |
| CD14-341 | neg | neg | | 0 | 0 |  |
| CD14-342 | neg | neg | | 0 | 0 |  |
| CD14-343 | neg | neg | | 0 | 0 |  |
| CD14-344 | neg | neg | | 0 | 0 |  |
| CD14-345 | pos | pos | | 17229.7 | 77056.3 |  |
| CD14-346 | pos | pos | | 97.4 | 328.2 |  |
| CD14-347 | neg | neg | | 0 | 0 |  |
| CD14-348 | neg | neg | | 0 | 0 |  |
| CD14-349 | neg | pos | | 4.6 | 0 | (NA) |
| CD14-350 | neg | neg | | 0 | 0 |  |
| CD14-351 | neg | neg | | 0 | 0 |  |
| CD14-352 | pos | pos | | 352.1 | 1611.0 |  |
| CD14-353 | neg | neg | | 0 | 0 |  |
| CD14-354 | pos | neg | | 0 | 0 |  |
| CD14-355 | pos | pos | | 251.7 | 771.9 |  |
| CD14-356 | neg | pos | | 203.3 | 711.1 | (1:10) pos |
| CD14-357 | pos | pos | | 498.0 | 2155.7 |  |
| CD14-358 | neg | neg | | 0 | 0 |  |
| CD14-359 | neg | neg | | 0 | 0 |  |
| CD14-360 | neg | neg | | 0 | 0 |  |
| CD14-361 | pos | pos | | 616.7 | 2157.7 |  |
| CD14-362 | neg | neg | | 0 | 0 |  |
| CD14-363 | neg | neg | | 0 | 0 |  |
| CD14-364 | neg | neg | | 0 | 0 |  |
| CD14-365 | neg | neg | | 0 | 0 |  |
| CD14-366 | neg | neg | | 0 | 0 |  |
| CD14-367 | neg | neg | | 0 | 0 |  |
| CD14-368 | neg | neg | | 0 | 0 |  |
| CD14-369 | neg | neg | | 0 | 0 |  |
| CD14-370 | neg | neg | | 0 | 0 |  |
| CD14-371 | neg | neg | | 0 | 0 |  |
| CD14-372 | neg | neg | | 0 | 0 |  |
| CD14-373 | pos | pos | | 634.7 | 1351.2 |  |
| CD14-374 | neg | neg | | 0 | 0 |  |
| CD14-375 | neg | neg | | 0 | 0 |  |
| CD14-376 | neg | neg | | 0 | 0 |  |
| CD14-377 | neg | neg | | 0 | 0 |  |
| CD14-378 | neg | neg | | 0 | 0 |  |
| CD14-379 | neg | pos | | 23.7 | 122.9 | (1:10) neg |
| CD14-380 | neg | neg | | 0 | 0 |  |
| CD14-381 | neg | neg | | 0 | 0 |  |
| CD14-382 | neg | neg | | 0 | 0 |  |
| CD14-383 | neg | neg | | 0 | 0 |  |
| CD14-384 | neg | neg | | 0 | 0 |  |
| CD14-385 | neg | neg | | 0 | 0 |  |
| CD14-386 | pos | pos | | 2467.4 | 7485.1 |  |
| CD14-387 | pos | pos | | 171.2 | 382.3 |  |
| CD14-388 | neg | neg | | 0 | 0 |  |
| CD14-389 | neg | neg | | 0 | 0 |  |
| CD14-390 | neg | neg | | 0 | 0 |  |
| CD14-391 | neg | neg | | 0 | 0 |  |
| CD14-392 | neg | neg | | 0 | 0 |  |
| CD14-393 | pos | pos | | 492.6 | 1140.8 |  |
| CD14-394 | pos | neg | | 0 | 0 |  |
| CD14-395 | pos | pos | | 18.3 | 43.1 |  |
| CD14-396 | neg | pos | | 96.6 | 23.9 | (1:10) neg |
| CD14-397 | pos | neg | | 0 | 0 |  |
| CD14-398 | pos | pos | | 949.9 | 1812.6 |  |
| CD14-399 | neg | neg | | 0 | 0 |  |
| CD14-400 | neg | neg | | 0 | 0 |  |
| CD14-401 | pos | pos | | 13099.6 | 53624.3 |  |
| CD14-402 | neg | neg | | 0 | 0 |  |
| CD14-403 | neg | neg | | 0 | 0 |  |
| CD14-404 | neg | pos | | 83.8 | 238.9 | (1:10) neg |
| CD14-405 | neg | neg | | 0 | 0 |  |
| CD14-406 | pos | pos | | 409.6 | 606.4 |  |
| CD14-407 | neg | neg | | 0 | 0 |  |
| CD14-408 | pos | pos | | 15837.8 | 29198.5 |  |
| CD14-409 | neg | neg | | 0 | 0 |  |
| CD14-410 | neg | neg | | 0 | 0 |  |
| CD14-411 | pos | pos | | 445.5 | 688.2 |  |
| CD14-412 | neg | neg | | 0 | 0 |  |
| CD14-413 | neg | neg | | 0 | 0 |  |
| CD14-414 | pos | pos | | 1009.0 | 1841.7 |  |
| CD14-415 | neg | neg | | 0 | 0 |  |
| CD14-416 | pos | neg | | 0 | 0 |  |
| CD14-417 | neg | neg | | 0 | 0 |  |
| CD14-418 | neg | neg | | 0 | 0 |  |
| CD14-419 | neg | neg | | 0 | 0 |  |
| CD14-420 | neg | neg | | 0 | 0 |  |
| CD14-421 | neg | neg | | 0 | 0 |  |
| CD14-422 | neg | neg | | 0 | 0 |  |
| CD14-423 | neg | neg | | 0 | 0 |  |
| CD14-424 | pos | neg | | 0 | 0 |  |
| CD14-425 | pos | pos | | 7966.4 | 21015.7 |  |
| CD14-426 | neg | neg | | 0 | 0 |  |
| CD14-427 | neg | neg | | 0 | 0 |  |
| CD14-428 | neg | neg | | 0 | 0 |  |
| CD14-429 | neg | neg | | 0 | 0 |  |
| CD14-430 | neg | neg | | 0 | 0 |  |

NA – not available

**Table S3**. Results of the evaluation of mock samples in field conditions used by lay or experienced laboratory personnel

|  |  | **Copies/swab** | **0** | | **160** | | **800** | | **3200** | | **16000** | |
| --- | --- | --- | --- | --- | --- | --- | --- | --- | --- | --- | --- | --- |
|  |  | Replicates | 1 | 2 | 1 | 2 | 1 | 2 | 1 | 2 | 1 | 2 |
| Laboratory | DjinniChip | Exp. User | neg | neg | pos | pos | pos | pos | pos | pos | pos | pos |
|  |  | Lay user | n.a. | n.a. | n.a. | n.a. | n.a. | n.a. | n.a. | n.a. | n.a. | n.a. |
|  | qPCR | Exp. User | neg | neg | pos | pos | pos | pos | pos | pos | pos | pos |
|  |  | Lay user | n.a. | n.a. | n.a. | n.a. | n.a. | n.a. | n.a. | n.a. | n.a. | n.a. |
| Field site 1: 33ºC, 24% humidity | DjinniChip | Exp. User | weak pos | neg | pos | pos | pos | pos | pos | pos | pos | pos |
|  |  | Lay user | pos | weak pos | very weak pos | pos | false neg | pos | pos | pos | pos | neg |
|  | qPCR | Exp. User | neg | neg | pos | pos | pos | pos | pos | pos | pos | pos |
|  |  | Lay user | neg | neg | neg | pos | pos | pos | pos | pos | pos | pos |
| Field site 2: 36ºC, 19% humidity | DjinniChip | Exp. User | weak pos | neg | pos | pos | pos | pos | pos | pos | pos | pos |
|  |  | Lay user | neg | weak pos | pos | weak pos | pos | pos | pos | pos | pos | pos |
|  | qPCR | Exp. User | neg | neg | neg | pos | pos | pos | pos | pos | pos | pos |
|  |  | Lay user | neg | neg | pos | neg | pos | pos | pos | pos | pos | pos |
| Field site 3: 25ºC, 50% humidity | DjinniChip | Exp. User | neg | neg | pos | pos | false neg | pos | pos | pos | pos | pos |
|  |  | Lay user | neg | false pos | pos | pos | pos | weak pos | pos | pos | pos | pos |
|  | qPCR | Exp. User | neg | neg | pos | pos | pos | pos | pos | pos | pos | pos |
|  |  | Lay user | neg | neg | pos | pos | pos | pos | pos | pos | pos | pos |
| Field site 4: 29ºC, 40% humidity | DjinniChip | Exp. User | false pos | false pos | pos | pos | pos | pos | pos | pos | pos | pos |
|  |  | Lay user | false pos | weak pos | pos | pos | pos | pos | pos | pos | pos | pos |
|  | qPCR | Exp. User | neg | neg | pos | pos | pos | pos | pos | pos | pos | pos |
|  |  | Lay user | neg | neg | pos | pos | pos | pos | pos | pos | pos | pos |

DjinniChip tests were performed by an experienced user (Exp.) and a lay user. qPCR was performed by a qualified operator using swab material eluted in the field by experienced and lay users. The results that did not correspond the sample status are highlighted
